# Supplementary material for: Impact of image averaging on vessel detection using optical coherence tomography angiography in eyes with macular oedema and in healthy eyes
Source: PLoS One. 2021 Oct 22;16(10):e0257859. doi: 10.1371/journal.pone.0257859 (PMC8535424; doi:10.1371/journal.pone.0257859)
Supplement: Attachment — Submitted filename: Comments OCTA image averaging.pdf [file pone.0257859.s002.pdf]

Impact of Image Averaging on Vessel Detection using Optical Coherence Tomography Angiography in Eyes with Macular Oedema and in Healthy Eyes"

1) The submitted manuscript investigates the reproducibility of vascular detection on averaged OCTA images.

Generally, the manuscript is well-presented for the purpose of the study. Other than a number of sentences that require shortening, and the frequently redundant use of articles, the manuscript is easy to read and understand.

The proposed methodology includes the necessary technical details; some additional information regarding the selection of contrast thresholds could probably be useful to the reader.

Statistical analysis was performed using the appropriate methods. Although the data support the ensuing conclusions, some additional comments/conclusions regarding the effect of pixel loss in reproducibility could be useful. After minor revision, the manuscript shall meet the criteria to be published in this journal.

2) Statistical analysis has been performed using the appropriate statistical methods and software. Data interpretation was performed using a widely accepted statistical software, and is fully representative of the implicated qualitative and quantitative variables.

3) The manuscript is presented in an intelligible fashion and written in standard English. There are some linguistic suggestions, which would maximize the reader's understanding; those are presented per manuscript section below. Generally, some of the sentences in the manuscript require shortening and use of a less complicated syntax, so as to facilitate the reader's grasp on a rather challenging topic.

ABSTRACT

— **Original text line**: *36-40*

Proposed changes: Please consider rephrasing this part in two separate, shorter sentences.

— **Original text line**: *42*

Proposed changes: Please define intra-class correlation coefficient, before using the abbreviation ICC.

INTRODUCTION

— **Original text line**: *58*

Proposed changes: Please avoid using two gerunds one after the other; for ex. you could replace assessing by the noun “assessment”

— **Original text line**: *66*

Proposed changes: “This process of averaging..”

— **Original text line**: *66-70*

Proposed changes: Please consider using the letter “the” less frequently; for ex. ~~the~~ quality, ~~the~~ speckle noise. Please consider this comment throughout the manuscript’s text.

— **Original text line**: *73-77*

Proposed changes: Please consider rephrasing this part.

— **Original text line**: *79*

Proposed changes: "..lead to loss of information".

RESULTS

— **Original text line**: *218-219*

Proposed changes: The authors should probably justify the use of non age-matched controls for the purpose of their study (in one sentence).

DISCUSSION

— **Original text line**: *351-355*

Proposed changes: Please consider rephrasing this part in two separate, shorter sentences.
